# The potential oral health impact of cost barriers to dental care: findings from a Canadian population-based study

**DOI:** 10.1186/1472-6831-14-78

**Published:** 2014-06-25

**Authors:** Brandy Thompson, Peter Cooney, Herenia Lawrence, Vahid Ravaghi, Carlos Quiñonez

**Affiliations:** 1 Discipline of Dental Public Health, Faculty of Dentistry, University of Toronto, Toronto, ON, Canada; 2 Office of the Canadian Oral Health Advisor, Public Health Agency of Canada, Ottawa, ON, Canada; 3 Oral Health & Society Research Unit, Faculty of Dentistry, McGill University, Montreal, QC, Canada

**Keywords:** Dental, Socioeconomic factors, Healthcare disparities, Dental care needs, Socio-demographic/economic factors, Health policy

## Abstract

**Background:**

Prior to the 2007/09 Canadian Health Measures Survey, there was no nationally representative clinical data on the oral health of Canadians experiencing cost barriers to dental care. The aim of this study was to determine the oral health status and dental treatment needs of Canadians reporting cost barriers to dental care.

**Methods:**

A secondary data analysis of the 2007/09 Canadian Health Measures Survey was undertaken using a sample of 5,586 Canadians aged 6 to 79. Chi square tests were conducted to test the association between reporting cost barriers to care and oral health outcomes. Logistic regressions were conducted to identify predictors of reporting cost barriers.

**Results:**

Individuals who reported cost barriers to dental care had poorer oral health and more treatment needs compared to their counterparts.

**Conclusions:**

Avoiding dental care and/or foregoing recommended treatment because of cost may contribute to poor oral health. This study substantiates the potential likelihood of progressive dental problems caused by an inability to treat existing conditions due to financial barriers.

## Background

The 2007/09 Canadian Health Measures Survey (CHMS) reports that the majority of dental care in Canada is privately financed and delivered on a fee-for-service basis, with 62.6 per cent of Canadians paying for dental care through employment-based insurance, 31.9 per cent through out-of-pocket payments, and 5.5 per cent through public funding. In turn, public dental care programs in Canada are generally only targeted to those that meet strict income eligibility criteria, such as those on social or disability assistance. Even among those that are eligible, coverage is typically for basic services and is largely limited to children and adolescents, and in most cases, only emergency treatment is provided to adults [1].

The public’s experience with the affordability of dental care continues as a topic of major interest to policy stakeholders in Canada. It is argued that a significant minority of the Canadian population is likely to experience financial barriers to accessing dental care, especially among those who do not have any form of dental insurance. One study reported that 26 per cent of Canadian adults deem dental care cost-prohibitive, with 35 per cent of them mentioning check-ups, cleanings and fillings as treatments they required but could not afford [2]. A study in 2009 collected data from working poor Canadian adults and demonstrated that almost 30 per cent of these individuals had been unable to afford dental care in the past, with 12.6 per cent of them reporting a competing need, having to sacrifice other spending (e.g. food) to pay for care [3]. In a national sample of Canadian adults, Locker et al. [4] demonstrated that 30 per cent reported avoiding or delaying dental visits, and 32.2 per cent reported not being able to receive all the treatment that was recommended due to cost.

In recent years, international studies have begun to highlight the impact that cost-prohibitive dental care needs can have on the health and general well-being of individuals. A survey conducted in the United Kingdom found that of the 43 per cent of respondents who reported avoiding the dentist due to cost, 26 per cent reported suffering long-term tooth decay, and 13 per cent reported suffering a periodontal abscess as a result [5]. An Australian study observed an inverse relationship between dental visiting frequency and Oral Health Impact Profile (OHIP-14) scores, which evaluates the consequences of oral conditions across various dimensions, such as functional limitation, physical pain, and psychological discomfort. Results showed that differences in mean OHIP-14 scores between groups with low and high dental visiting patterns was greater than two-fold, indicating worse oral conditions among those who were unable to visit a dentist in a given year [6]. Canadian authors, Locker et al. [4] used a more direct analysis, and demonstrated that those reporting cost barriers to accessing dental care also reported worse oral health outcomes after controlling for private insurance coverage, household income, sex, age and education. Their results showed that the extent and severity of OHIP-14 scores increased alongside the number of positive responses to cost barrier questions. Most recently, Ramraj et al. [7] showed that over a third of Canadians require dental treatment, with those who report cost barriers to dental care being 2.7 times more likely to have an unmet dental care need.

Prior to the 2007–09 CHMS, there was no nationally representative clinical data on the oral health of Canadians experiencing cost barriers to dental care. The availability of this new data provides an opportunity to explore these barriers and their potential consequences. Thus, the aim of this study was to determine the oral health status and dental treatment needs of Canadians reporting cost barriers to dental care.

## Methods

Data from the 2007/09 CHMS, conducted by Statistics Canada in partnership with Health Canada and the Public Health Agency of Canada, were utilized for this study. The CHMS is the first nationally representative study on clinically measured oral health conditions in Canada since the Nutrition Canada Survey of the early 1970s. The purpose of the CHMS was to collect information “to help evaluate the extent of health problems [and] to ascertain relationships among disease risk factors, health protection practices, and health status based on direct measures” [8].

To access the confidential microdata files of the CHMS, the principal applicant for this study was required to submit a proposal to the Social Sciences and Humanities Research Council (SSHRC) and Statistics Canada. The principal applicant conducted all statistical analyses at Statistics Canada’s Toronto Research Data Center (RDC) under the provisions of the Statistics Act in accordance with Statistics Canada’s confidentiality rules. Confidential microdata at the RDC are accessible only to researchers with approved projects who have been sworn in under the Statistics Act as ‘deemed employees’.

### Sample & study design

A total of 5,604 people living in privately occupied dwellings across Canada were surveyed representing approximately 97 percent of the Canadian population between 6 and 79 years of age. Further details pertaining to the CHMS study design, sample and data collection have been published elsewhere [8].

### Data collection

Data collection was conducted in two steps. First, a questionnaire was administered in respondent’s homes seeking information on socio-demographic characteristics, oral health, oral symptoms, and oral care habits including dental care utilization patterns [8]. Consent was implied when agreeing to respond to the questions. Next, a clinical examination in a mobile clinic was conducted by calibrated examining dentists supplied by the Canadian Forces. The dentist-examiner asked respondents questions relating to dental symptoms (pain, bleeding, etc.) and medical history questions to ensure eligibility [8]. Written consent was obtained for the clinical examination [9].

### Variables of interest

#### Cost barriers

Two variables were used to measure cost barriers to dental care, corresponding to two questions with a “yes” or “no” response: “In the past 12 months, have you avoided going to a dental professional because of the cost of dental care?” and “In the past 12 months, have you avoided having all the dental treatment that was recommended because of the cost?”

#### Oral health status

Oral health variables used in this study include perceived oral health (how individuals view their health) and clinically evaluated oral health (how health professionals determine health status and one’s need for care).

#### Self-perceived oral health

Self-perceived oral health includes self-reported oral health and self-reported oral pain. For self-reported oral health, respondents were asked to rate the health of their mouth using the categories excellent, very good, good, fair, and poor. These categories were dichotomized into “excellent to good” and “fair to poor”. For self-reported oral pain, respondents reported whether they experienced pain in their mouth based on the following question: “In the past 12 months, how often have you had any other persistent or on-going pain anywhere in your mouth?” Answers were dichotomized into categories “often and sometimes” and “rarely or never”.

#### Clinically evaluated oral health

##### Decayed, missing and filled teeth

The number of decayed, missing and filled teeth (deft and DMFT) were clinically determined and recorded for each tooth crown for both adults and children. The total DMFT value is a continuous outcome variable and is a combined deft and DMFT figure. That is, decayed, missing and filled teeth for both primary and permanent dentitions were added to give a combined value for each respondent.

##### Treatment needs

During the clinical examination, the dentist examiner determined recommendations for the type(s) of treatment needed for each participant. The quantity of needs within each treatment category could not be specified since recommendations for needing treatment were categorized as either a “yes” or “no”.

The following treatment categories were used in this study: prevention (i.e. examination, prophylaxis, fluoride, sealants, radiographs); restorative (i.e. fillings, crowns, bridge for restoration of carious lesions); surgery; periodontal (i.e. scaling, root planning, periodontal surgery); endodontic (i.e. root canal therapy); prosthodontic (i.e. removable/fixed, partial/full dentures, implant, bridge or crown); and urgent (i.e. treatment needed within a week; includes urgent problems from all treatment categories).

### Analyses

Survey weights were used to ensure data were nationally representative. Each weight corresponded to the number of people represented by the survey respondent in the population as a whole. In addition, bootstrap weights were applied to take into account of the CHMS’s complex, multi-stage sampling design.

Descriptive frequencies were calculated to observe the socioeconomic and demographic characteristics of each sample. Chi square tests were conducted to test the association between reporting cost barriers to care and oral health outcomes.

Logistic regressions were conducted for each outcome variable to determine which factors were the strongest predictors of reporting cost barriers. The crude and adjusted odds ratios, 95 percent confidence intervals (CIs) and P-values were recorded. The significance level was set at P < 0.05.

All statistical analyses were completed using STATA v.12. Missing data were the result of non-response to some or all questions in the survey. These data were coded by Statistics Canada prior to analyses and were removed from the data set. In addition, respondents who did not attend the dental examination were excluded from the analyses (n = 18). As part of the disclosure process at Statistics Canada’s Research Data Centre, unweighted proportions and counts were not permitted for release. Only weighted data are presented in this study.

## Results

Information on the CHMS sample characteristics, including socioeconomic and demographic characteristics can be found in Health Canada’s report on the findings of the oral health component of the CHMS [8]. Nearly one in five (23 per cent) of those surveyed reported experiencing a cost barrier to dental care, whether it was avoiding a dentist, declining recommended dental treatment, or both. Approximately 17.3 per cent (CI: 14.7, 20.3) of those surveyed reported avoiding a dental professional due to cost and 16.5 per cent (CI: 15.0, 18.2) reported declining recommended dental treatment due to cost.

Respondents aged 20 to 39 (23.7%, CI: 19.1, 29.0), females (19.2%, CI: 16.1, 22.7), those without dental insurance (35.9%, CI: 30.4, 41.9) and from the lowest income category (35.2%, CI: 27.1, 44.3) reported avoiding a dental professional in the last year due to cost most often. Similarly, respondents aged 20 to 39 (19.4%, CI: 16.4, 22.7), females (18.6%, CI: 16.9, 20.4), those without dental insurance (27.4%, CI: 23.1, 32.1) and from the lowest income category (31.6%, CI: 24.7, 39.3) reported declining recommended dental treatment in the last year due to cost. Further details, including the breakdown of socioeconomic and demographic characteristics of those reporting cost barriers to dental care are published elsewhere [10].

### Self-perceived oral health

**Figure 1:**
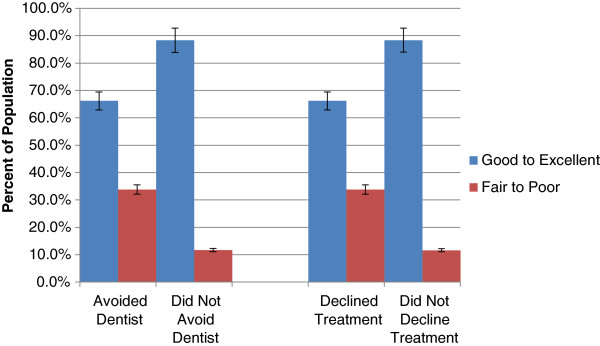
Self-reported oral health and cost barriers to dental care, 2007–09.

**Figure 2:**
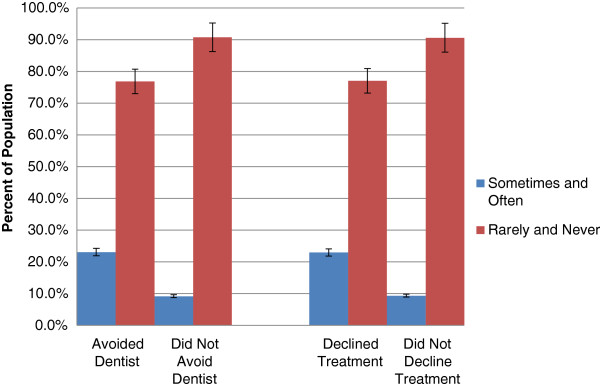
Self-reported oral pain and cost barriers to dental care, 2007–09.

A greater proportion of individuals who reported avoiding a dental professional due to cost also reported having fair to poor oral health (33.8%, CI: 27.8, 40.4) compared to those who did not report avoiding a dental professional due to cost (11.7%, CI: 9.9, 13.7) (Figure 1). Similarly, a greater proportion of individuals who reported declining recommended dental treatment due to cost also reported having fair to poor oral health (33.8%, CI: 27.8, 40.4) compared to their counterparts (11.6%, CI: 10.0, 13.4) (Figure 1).A greater proportion of individuals who reported avoiding a dental professional due to cost reported having oral pain sometimes or often (23.1%, CI: 19.4, 27.3), compared to their counterparts (9.2%, CI: 7.9, 10.7). Similarly, a greater proportion of individuals who reported declining recommended dental treatment due to cost reported having oral pain sometimes or often (23.0%, CI: 19.1, 27.4) compared to their counterparts (9.4%, CI: 8.0, 10.9) (Figure 2).

### Clinically evaluated oral health

#### Decayed, missing and filled teeth

A greater proportion of individuals who reported avoiding a dental professional due to cost had experienced dental caries in their lifetime compared to their counterparts (93.5%, CI: 0.91, 0.95 vs. 87.6%, CI: 0.86, 0.90) (Table 1). They had over three times the amount of untreated decay, with a mean decay score of 1.37 (CI: 0.98, 1.77), compared to 0.37 (CI: 0.29, 0.45) among their counterparts.

**Table 1:** Mean DMFT and cost barriers to dental care, 2007-09

	**Avoided Dentist due to cost (95% CI)**	**Did not avoid dentist due to cost (95% CI)**	**Declined treatment due to cost (95% CI)**	**Did not decline treatment due to cost (95% CI)**
**Mean D**	1.37 (0.98,1.77)	0.37 (0.29,0.45)	1.18 (0.79,1.56)	0.42 (0.33,0.51)
**Mean M**	2.22 (1.86,2.58)	1.61 (1.43,1.79)	2.44 (2.12,2.78)	1.57 (1.42,1.72)
**Mean F**	5.98 (5.32,6.63)	6.80 (6.44,7.17)	6.95 (6.12,7.80)	6.60 (6.27,6.93)
**Mean DMFT**	9.57 (8.77,10.37)	8.78 (8.38,9.18)	10.57 (9.91,11.24)	8.58 (8.23,8.94)

Similarly, more individuals who declined recommended treatment due to cost had experienced dental caries in their lifetime compared to their counterparts (93.9%, CI: 91.3, 95.8 vs. 87.6%, CI: 85.7, 89.2) (Table 1). In addition, they had nearly three times the amount of untreated decay, with a mean decay score of 1.18 (CI: 0.79, 1.56), compared to 0.42 (CI: 0.33, 0.51) among their counterparts.

#### Treatment needs

**Figure 3:**
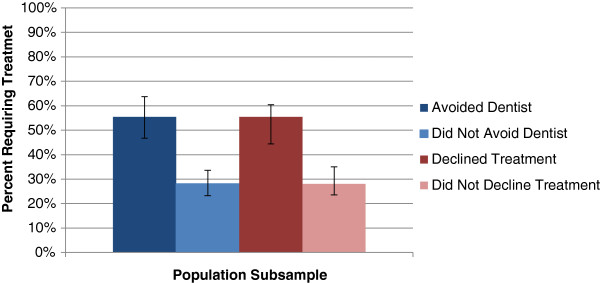
Proportion with a clinical treatment need among Canadians reporting cost barriers to dental care, 2007–09.

The proportion of individuals with a treatment need was much higher for those reporting cost barriers to dental care (Figure 3). Approximately 55 per cent (55.4% CI: 46.7, 63.8) of those who avoided a dental professional due to cost were clinically determined to have a dental treatment need compared to only 28 per cent (28.1% CI: 23.2, 33.7) among their counterparts. A greater proportion of those avoiding due to cost also had multiple treatment needs (33.9%, CI: 28.0, 40.3 vs. 12.7%, CI: 9.0, 17.4).

There was a general trend that those who avoided a dentist had a higher prevalence of needing treatment; more than double in all treatment need categories (with the exception of urgent needs). Restorations were needed most with 37.7 per cent (CI: 31.8, 44.0) of respondents requiring fillings, compared to only 16.8 per cent (CI: 14.0, 20.0) among those who did not report avoiding due to cost (Table 2).The proportion of Canadians with a treatment need was also much higher for those reporting declining recommended dental treatment due to cost (Figure 3). Approximately 55 per cent (55.4%, CI: 44.4, 60.5) of those who declined treatment were clinically determined to have a dental treatment need compared to only 28 per cent (28.1%, CI: 23.6, 35.0) among their counterparts. A greater proportion of those declining treatment due to cost also had multiple treatment needs (29.4%, CI: 22.2, 37.8 vs. 13.7%, CI: 9.8, 18.9).

**Table 2:** Type of treatment needed by Canadians who reported cost barriers to dental care in the last year, 2007-09

	**Avoided dentist due to cost (%, 95% CI)**	**Did not avoid dentist due to cost (%, 95% CI)**	**Declined treatment due to cost (%, 95% CI)**	**Did not decline treatment due to cost (%, 95% CI)**
**Prevention**	27.4 (20.9, 35.1)	10.9 (7.9, 14.9)	21.1 (15.0, 28.9)	12.3 (9.0, 16.6)
**Restorative**	37.7 (31.8, 44.0)	16.8 (14.0, 20.0)	36.9 (30.0, 44.4)	17.2 (14.2, 20.6)
**Surgery**	15.8 (12.2, 20.1)	5.7 (4.1, 7.8)	15.9 (11.7, 21.3)	5.8 (4.1, 8.2)
**Periodontal**	9.6 (6.7, 13.5)	4.1 (2.8, 6.1)	8.0 (6.1, 10.4)	4.5 (3.0, 6.6)
**Endodontic**	6.1 (3.9, 9.4)	1.1 (0.6, 2.1)	6.0 (3.3, 10.7)	1.2 (0.7, 1.9)
**Prosthodontic**	16.2 (13.1, 19.8)	8.4 (6.2, 11.3)	15.7 (11.0, 21.9)	8.6 (6.6, 11.1)
**Urgent**	5.7 (3.4, 9.5)	5.9 (2.5, 13.4)	6.7 (3.2, 13.6)	5.6 (2.8, 10.9)

Similarly, there was a trend that those who declined treatment due to cost also had a higher prevalence of needing treatment (with the exception of urgent needs). Restorations were also needed most with 36.9 per cent (CI: 30.0, 44.4) of respondents requiring fillings, compared to only 17.2 per cent (CI: 14.2, 20.6) among their counterparts (Table 2).

### Predictors of cost barriers

Tables 3 and 4 exhibit the results of the logistic regression analyses. After controlling for socioeconomic and demographic variables, respondents with untreated decay were 1.1 times more likely to report avoiding a dental professional in the last year due to cost (CI: 1.02, 1.2, P = 0.021) and 1.1 times more likely to decline recommended dental treatment in the last year due to cost (CI: 1.02, 1.2, P = 0.018). Further, individuals who reported having fair to poor oral health were 3.1 times more likely to avoid a dental professional due to cost compared to those that reported having good to excellent oral health (CI: 2.1, 4.5, P = 0.001) and 3.0 times more likely to decline recommended dental treatment due to cost compared to those reporting good to excellent oral health (CI: 2.3, 3.9, P = 0.001).

**Table 3:** Predictors for avoiding a dental professional in the past year due to cost, 2007/09

**Variables**	**Unadjusted odds ratio (95% CI)**	**P-value**	**Adjusted odds ratio (95% CI)**	**P-value**
Age*				
6-19 (reference)				
20-39	2.41 (1.73,3.35)	0.001	2.46 (1.18,5.12)	0.021
40-59	1.65 (1.09,2.48)	0.021	1.76 (1.09,2.83)	0.025
**Sex**				
Males (reference)				
Females	1.29 (1.01,1.67)	0.013	1.43 (1.00,2.05)	0.048
**Immigrant status**				
Non-Immigrant (reference)				
Immigrant	1.59 (1.20,2.12)	0.004	1.19 (0.75,1.88)	0.417
**Education**				
> High school (reference)				
< High school	0.82 (0.66,1.03)	0.770	0.85 (0.54,1.34)	0.451
**Employment status**				
Full-time employed (reference)				
Part-time employed	1.29 (0.72,2.29)	0.352	0.80 (0.40,1.60)	0.495
Unemployed	1.35 (1.11,1.64)	0.006	0.90 (0.59,1.36)	0.591
**Income**				
Higher (reference)				
Upper middle	2.51 (1.62,3.91)	0.001	1.82 (1.18,2.80)	0.011
Lower middle	5.40 (3.38,8.62)	0.001	3.79 (2.16,6.67)	0.001
Lower	5.64 (3.56,8.93)	0.001	4.27 (1.69,10.74)	0.005
**Insurance**				
Private (reference)				
Public	1.04 (0.49,2.18)	0.918	0.42 (0.12,1.56)	0.175
None	5.95 (4.60,7.70)	0.001	5.85 (4.20,8.15)	0.001
**Self-reported oral health**				
Good to excellent (reference)				
Fair to poor	3.88 (2.74,5.48)	0.001	3.09 (2.11,4.54)	0.001
**Oral health**				
D (decayed teeth)	1.30 (1.19,1.41)	0.001	1.12 (1.02,1.23)	0.021
M (missing teeth)	1.05 (1.01, 1.10)	0.014	1.02 (0.97,1.08)	0.343

*Age groups 6–11 and 12–19 were combined for the logistic regression analyses since certain data was not collected for respondents aged 6–11 (i.e. income and employment status).

**Table 4:** Predictors for declining recommended dental treatment in the past year due to cost, 2007/09

**Variables**	**Unadjusted odds ratio (95% CI)**	**P-value**	**Adjusted odds ratio (95% CI)**	**P-value**
Age*				
6-19 (reference)				
20-39	2.87 (1.98,4.16)	0.001	1.85 (1.00,3.43)	0.050
40-59	2.75 (1.67,4.52)	0.001	1.83 (1.03,3.25)	0.042
**Sex**				
Males (reference)				
Females	1.36 (1.09,1.69)	0.011	1.47 (1.10,1.97)	0.015
**Immigrant status**				
Non-Immigrant (reference)				
Immigrant	1.15 (0.76,1.75)	0.479	1.43 (1.05,1.95)	0.028
**Education**				
> High school (reference)				
< High school	0.63 (0.39,1.04)	0.068	0.65 (0.05,0.82)	0.002
**Employment status**				
Full-time employed (reference)				
Part-time employed	1.01 (0.52,1.97)	0.964	1.37 (0.85,2.22)	0.176
Unemployed	0.83 (0.66,1.05)	0.104	1.23 (0.96,1.57)	0.094
**Income**				
Higher (reference)				
Upper middle	2.05 (1.58,2.66)	0.001	1.61 (1.25,2.08)	0.002
Lower middle	3.74 (2.49,5.60)	0.001	2.95 (1.69,5.15)	0.001
Lower	4.21 (2.73,6.50)	0.001	2.64 (1.59,4.38)	0.001
**Insurance**				
Private (reference)				
Public	1.81 (0.99,3.30)	0.054	1.21 (0.49,2.97)	0.655
None	3.08 (2.39,3.99)	0.001	2.35 (1.62,3.40)	0.001
**Self-reported oral health**				
Good to excellent (reference)				
Fair to poor	4.11 (3.18,5.3)	0.001	3.04 (2.34,3.94)	0.001
**Oral health**				
D (decayed teeth)	1.09 (1.02,1.17)	0.018	1.20 (1.13,1.28)	0.001
M (missing teeth)	1.04 (0.99,1.10)	0.140	1.07 (1.05,1.1)	0.001

*Age groups 6–11 and 12–19 were combined for the logistic regression analyses since certain data was not collected for respondents aged 6–11 (i.e. income and employment status).

## Discussion

Avoiding dental care because of cost represents a barrier that is present prior to seeking care, while foregoing recommended dental treatment due to cost occurs when, after making an initial visit, cost prevents one from proceeding with recommended care. Both of these circumstances suggest the potential for progressive damage to teeth or the worsening of oral health due to cost barriers [4,5,7,11]. The aim of this study was to determine the oral health status and dental treatment needs of Canadians reporting cost barriers to dental care and it was demonstrated that over one in five Canadians reported barriers. These individuals had more untreated decay, missing teeth, and reported having poorer oral health and oral pain more often. It was also found that those reporting cost barriers had a higher prevalence of needing dental treatment and had more treatment needs. Additionally, having untreated decay was found to be predictive of reporting financial barriers to care, suggesting the likelihood of negatively progressing dental conditions related to the inability to secure treatment based on cost barriers to dental care.

These findings support results from longitudinal research demonstrating that routine dental attendance results in better oral health outcomes, including fewer missing teeth [12,13], fewer decayed teeth [12,13], lower overall DMFS (decayed, missing, and filled surfaces) scores [12,13], better oral health-related quality of life [13-17] and better self-reported oral health [13,16]. Within the limitations of a cross-sectional study, and based on previous longitudinal findings, we can infer that once financial barriers are removed, the oral health of Canadians reporting cost barriers to care have the potential to improve.

By diminishing financial barriers to care, the overall burden of oral disease at the societal level may be reduced, and may contribute to a healthier, more productive society. Oral diseases are relatively straightforward to treat and deliver short-term benefits in reducing the signs of inflammation [18] related to systemic adverse health conditions such as diabetes [19-21] and cardiovascular disease [22,23]. Therefore, in addition to the importance of good oral health to improve quality of life [24-27], it is likely that reducing the inflammatory burden arising from oral disease may also reduce the cumulative systemic inflammatory burden on the body [18]. The results of oral disorders can also be felt not only physically and socially but also economically. Given that cardiovascular disease alone costs the Canadian economy more than $20.9 billion every year [28], reducing the oral health impact on systemic disease could have positive economic influences. Additionally, oral health outcomes (i.e. DMFT, periodontal disease, etc.) have been linked to the social impacts of disease on daily life, including time loss from work, school or normal activities. Hayes et al. [29] found that over 40 million hours are lost annually due to dental problems in Canada, resulting in subsequent potential productivity losses of over $1 billion dollars. Thus, cost barriers to dental care act as a catalyst for dental-related time loss from work and school, ultimately contributing productivity losses at a societal level.

In a privately financed dental care system like Canada’s, dental insurance mitigates the potential barrier of upfront costs, meaning that the insured reported cost barriers much less often than the uninsured. Recent research shows that, even after controlling for other factors, including income, the uninsured were almost six times more likely to avoid the dentist because of cost compared with the insured [10]. The need for policies aimed at controlling the costs of dental services, and increasing their affordability for vulnerable groups is apparent. In terms of affordability, from a policy perspective, income and insurance are queen and king. In the current economic and political environment, it is likely that more can be done to provide insurance than increasing wages or improving income redistribution, for example. Thus, in order to reduce cost barriers to care and potentially improve oral health outcomes, there is a need to improve the quality of dental insurance coverage, or to ensure that cost-sharing arrangements be kept low and that important services are not excluded from insurance plans. This is evidenced by The RAND Health Insurance Experiment, a large-scale study of health care costs, utilization and outcomes in the United States [30] which early on confirmed the importance of affordable dental insurance to oral health, by demonstrating that a reduction in cost-sharing for dental services actually improved oral health, especially for subgroups of the population with the poorest oral health outcomes.

Unfortunately, the participation of employers in providing employment-based insurance in Canada has declined over the past decades [31,32]. The continual and invariant increase in the costs of dental care has contributed to the increasing costs of dental plans. These costs ultimately fall back onto employers and have continued to rise at well beyond the rate of inflation [32]. In response, the private dental insurance system in Canada is gradually becoming unsustainable since the costs of coverage have continued to outpace the purchasing power of many employers as the main payer for insurance benefits. In addition to the prevailing role of employer-employee contracts, inadequate financial support from governments in reducing barriers to dental care have fostered an environment where access to care is now more strongly associated with one’s level of income and insurance than ever before [4,31]. Much of this speaks to the “inverse care law”, where people that need the most care tend to receive the least [33], a term that has been used to describe the dental care situation in Canada. Given the dramatic increases in the costs of providing dental benefits, in conjunction with economic challenges, we postulate that the number of underinsured and uninsured individuals will continue to increase [31,32,34].

Provincial governments have begun to extend affordable dental insurance to uninsured children; however, the unmet oral health needs of uninsured adults continue to be ignored in the health care system without a cohesive political response by provincial governments. This has important impacts, as one could argue that the deterioration of private and public dental benefits coverage for adults has contributed to the use of acute health care settings for basic dental problems. Quiñonez et al. [35] demonstrated that most dental-related emergency visits are non-urgent, preventable and often result in an intervention that does not provide a definitive solution to the dental problem (e.g. pharmacotherapy). Further, it was found that the majority of individuals visiting the emergency room for dental care are low income and ineligible for public funding, such as the working poor, seniors, or those on social assistance.

Overall, improving oral health outcomes requires targeted investments in programs and services that match the needs of the public and that target financial constraints. It is important for governments to consider policies that attempt to control the costs of health care plans and contribute to plans for vulnerable populations. These policies may, for example, include mandating, through legislation, the presence of health care benefits in all employment-employee contracts.

### Strengths and limitations

While self-reported data are the most convenient and readily attained method for assessing oral health outcome information, it has been shown to be influenced by one’s culture, personal beliefs, and other social factors, such as age, education and income [36]. Thus, this study makes an important contribution by highlighting the oral health status of Canadians who reported experiencing cost barriers to dental care by examining their clinical oral health status and needs, as determined by calibrated dentists. Further, this study provides valuable baseline information for future studies to assess whether financial barriers to dental care are getting better or worse in Canada.

This study has several limitations, specifically the inability to make causal inferences based on the cross-sectional nature of the data and shortcomings in breadth and detail of the variables. For example, it is impossible to know whether the cost barriers caused poorer oral health outcomes. Lastly, within the confines of the data collected, it is not possible to understand what treatments were considered unaffordable. This is important information to know, particularly from a public health perspective, where the inability to afford basic restorative services is much different than not being able to afford orthodontic services, for example. Lastly, assumptions were made when analyzing the two cost barrier questions. For example, the first question, *“In the past 12 months, have you avoided going to a dental professional because of the cost of dental care?”* assumes that the respondent avoided the dentist altogether due to cost. For the second question, “*In the past 12 months, have you avoided having all the dental treatment that was recommended because of the cost?”* it is assumed that the respondent visited a dentist, was recommended treatment, and then declined the treatment due to cost. For respondents who answered “yes” to both questions, it is assumed that they had experienced both situations in the same year, on separate occasions.

## Conclusion

This is the first study of its kind in Canada, and makes an important contribution by highlighting the oral health of Canadians who report cost barriers to dental care. This study demonstrated that cost barriers to care may impact oral health, establishing that Canadians who report cost barriers to dental care have more dental treatment needs, more dental decay and more missing teeth. This substantiates the potential likelihood of progressive dental problems caused by an inability to treat existing conditions due to financial barriers.

## Abbreviations

CHMS: Canadian Health Measures Survey; OHIP-14: Oral Health Impact Profile; DMFT: Decayed, missing and filled teeth; DMFS: Decayed, missing, and filled surfaces.

## Competing interests

The authors declare that they have no competing interests.

## Authors’ contributions

BT, PC, HL, VR and CQ contributed to the conception and design of the study. BT was granted permission to access Statistics Canada’s Research Data Center in Toronto in order to analyze and interpret the data. BT drafted the manuscript. VR provided statistical assistance and interpretation of data. CQ revised the manuscript critically for important intellectual content. All authors read and approved the final manuscript.

## Pre-publication history

The pre-publication history for this paper can be accessed here:

http://www.biomedcentral.com/1472-6831/14/78/prepub
